# Prospective randomized study for optimal insulin therapy in type 2 diabetic patients with secondary failure

**DOI:** 10.1186/1475-2840-7-16

**Published:** 2008-05-29

**Authors:** Yumi Miyashita, Rimei Nishimura, Masami Nemoto, Toru Matsudaira, Hideaki Kurata, Tamotsu Yokota, Kuninobu Yokota, Katsuyoshi Tojo, Kazunori Utsunomiya, Naoko Tajima

**Affiliations:** 1 Division of diabetes, Metabolism and Endocrinology, Department of Internal Medicine, Jikei University School of Medicine, Tokyo, Japan

## Abstract

**Background:**

The large clinical trials proved that Basal-Bolus (BB) insulin therapy was effective in the prevention of diabetic complications and their progression. However, BB therapy needs multiple insulin injections per a day. In this regard, a biphasic insulin analogue needs only twice-daily injections, and is able to correct postprandial hyperglycemia. Therefore it may achieve the blood glucose control as same as that of BB therapy and prevent the diabetic complications including macroangiopathy.

**Methods:**

In PROBE (Prospective, Randomized, Open, Blinded-Endpoint) design, forty-two type 2 diabetic patients (male: 73.8%, median(inter quartile range) age: 64.5(56.8~71.0)years) with secondary failure of sulfonylurea (SU) were randomly assigned to BB therapy with a thrice-daily insulin aspart and once-daily basal insulin (BB group) or to conventional therapy with a twice-daily biphasic insulin analogue (30 Mix group), and were followed up for 6 months to compare changes in HbA1c, daily glycemic profile, intima-media thickness (IMT) of carotid artery, adiponectin levels, amounts of insulin used, and QOL between the two groups.

**Results:**

After 6 months, HbA1c was significantly reduced in both groups compared to baseline (30 Mix; 9.3(8.1~11.3) → 7.4(6.9~8.7)%, p < 0.01, vs BB;8.9(7.7~10.0) → 6.9(6.2~7.3)%, p < 0.01), with no significant difference between the groups in percentage change in HbA1c (30 Mix; -14.7(-32.5~-7.5)% vs BB -17.8(-30.1~-11.1)%, p = 0.32). There was a significant decrease in daily glycemic profile at all points except dinner time in both groups compared to baseline. There was a significant increase in the amount of insulin used in the 30 Mix group after treatment compared to baseline (30 Mix;0.30(0.17~0.44) → 0.39(0.31~0.42) IU/kg, p = 0.01). There was no significant difference in IMT, BMI, QOL or adiponectin levels in either group compared to baseline.

**Conclusion:**

Both BB and 30 mix group produced comparable reductions in HbA1c in type 2 diabetic patients with secondary failure. There was no significant change in IMT as an indicator of early atherosclerotic changes between the two groups. The basal-bolus insulin therapy may not be necessarily needed if the type 2 diabetic patients have become secondary failure.

**Trial registration:**

Current Controlled Trials number, NCT00348231

## Background

The goal of diabetes management consists in the prevention of diabetic complications, as well as their progression, by achieving favorable control over glycemic and other risk factors [1]. Insulin regimens currently in use include conventional once- or twice-daily insulin injection therapy, and intensive insulin therapy with a rapid-acting insulin analogue administered three times daily before meals to reproduce the physiologic insulin secretion dynamics seen in healthy individuals complemented by Neutral Protamine Hargedorn (NPH) or long-acting insulin analogue injections administered at night on an on-demand basis.

Findings from large-scale epidemiological studies, such as the Diabetes Control Complication Trial (DCCT) [2,3] and the Kumamoto Study [4], clearly demonstrate that intensive insulin therapy significantly reduces the incidence of diabetic complications as well as prevents disease progression in type 1 and type 2 diabetic patients compared to conventional insulin therapy.

However, an international meeting jointly held by the WHO and Juvenile Diabetes Foundation International in 1985 pointed to a need for insulin formulations that could be administered immediately before meals, in contrast to the rapid-acting insulin formulations that needed to be administered 30 minutes before meals, with a blood concentration profile that could mimic the dynamics of insulin secretion that occur postprandially in healthy individuals [5]. This led to the insulin aspart and insulin Lispro being developed and approved for clinical use in the 1990's.

While intensive insulin therapy using these formulations offered more physiologic and rigorous glycemic control than with conventional insulin formulations, the use of these formulations involved multiple injections, thus often leading to poor patient acceptance of the treatment offered, as well as to some injections being missed.

In the late 1990s, it was demonstrated through the DECODE study [6] and the Funagata study [7] that high 2-hour glucose levels after an oral glucose tolerance test (OGTT) are associated with increased risk for cardiovascular disease compared to high fasting glucose levels, which led to more rigorous correction of postprandial hyperglycemia being called for in the management of diabetes.

A biphasic insulin analogue preparation was approved and became available overseas in 2000 and in Japan in 2003. This biphasic insulin analogue consists of 30% free and 70% protamine-bound insulin aspart, and is expected with its twice-daily pre-meal injections to improve patient compliance to insulin injection therapy as well as the quality of life (QOL) of patients given the treatment, while at the same time improving postprandial hyperglycemia better than conventional mixed preparations, thus potentially reducing the risk for cardiovascular disease.

In type 1 diabetes in which endogenous insulin secretion is completely depleted, basal-bolus insulin therapy with an ultra rapid-acting insulin analogue represents an optimal therapeutic choice as it closely mimics physiologic insulin secretion. In contrast, in type 2 diabetic patients whose endogenous insulin secretion is preserved to a certain extent, however, twice-daily insulin injection therapy using a biphasic insulin analogue may be able to produce comparable glycemic control to that achieved with basal-bolus therapy using an ultra rapid-acting insulin analogue, thus potentially commending itself as viable a therapeutic option as basal-bolus therapy in preventing diabetic complications, including macrovascular complications.

Furthermore, it was also assumed that biphasic insulin analogues might have a role in facilitating insulin therapy for type 2 diabetes in the clinical setting, thus contributing to overall improved QOL with insulin therapy.

The aim of this study was the comparison of efficacy between a twice-daily insulin regimen using a biphasic insulin analogue regimen and basal-bolus regimen using thrice-daily ultra rapid-acting insulin analogue in these patients in type 2 diabetic patients with secondary failure of sulfonylureas(SU). Furthermore, intima-media thickness (IMT) [8] was measured in all patients given these regimens to compare their efficacy in preventing macrovascular complications, with adiponectin levels and patient QOL also compared between the treatment groups.

## Methods

### Subjects and Methods

This study was a single-center study with a prospective, randomized, open, blinded-endpoint evaluation (PROBE) design in type 2 diabetic patients being treated on an outpatient basis at our clinic, who ranged in age between 20 years of age or older but younger than 80 years old, and who had secondary failure. Secondary failure was defined as patients who even were receiving maximally tolerated doses of sulfonylurea and failed to achieve HbA1c < 8%. The study subjects were randomly assigned to twice-daily insulin therapy with BIAsp70/30 (NovoRapid 30 Mix^®^), a biphasic insulin analogue formulation given twice daily immediately before breakfast and dinner (30 Mix group), or to basal-bolus insulin therapy with insulin aspart (NovoRapid^®^) in combination with NPH insulin injected at night on an on-demand basis (BB group). A computer-assisted, least square method was used in random assignment to ensure that there was no baseline difference in gender, age, BMI, HbA1c, or duration of SU use between the two groups.

Prior to starting the subjects on insulin therapy, all SU was discontinued in the subjects, while all insulin-sensitizing agents (metformin or thiazolidinedione derivatives) being concurrently used with SU in the subjects were continued. All α-glucosidase inhibitors or phenylalanine derivatives being concurrently used with SU were discontinued. Use of any new oral hypoglycemic agents (OHA) in the subjects was not allowed during follow-up.

All subjects agreed to self-measuring their blood glucose levels (SMBG) at home during follow-up after initiation of insulin therapy.

At initiation of insulin therapy, all subjects were hospitalized and their insulin dose was adjusted at the discretion of the attending physician every 2 to 3 days for the first week of treatment in an attempt to achieve fasting glucose levels less than 130 mg/dl and 2-hour postprandial glucose levels less than 180 mg/dl as per the definition of "favorable glycemic control" given in the Japanese Guidelines for the Management of Diabetes by Japan Diabetes Society. After hospital discharge, the subjects were asked to visit the outpatient clinic once a month, when the attending physician adjusted the insulin dose with the goal of achieving HbA1c < 6.5% in mind, in light of the self-measured glucose levels reported by the subjects.

### Laboratory procedures

Parameters evaluated at hospital admission included height, body weight, blood pressure after resting, HbA1c, daily glycemic profile (in terms of 7-point glucose measurements; immediately before and 120 minutes after breakfast, lunch, and dinner, at bedtime), total amount of insulin used, and adiponectin levels, and these parameters were evaluated once again after 24 weeks of treatment to allow comparison between the treatment groups for changes in these variables.

### Carotid B-Mode Ultrasound

Ultrasonographic scanning of carotid artery was performed at the beginning and the end of the study. A series of ultrasonographic scans was performed using an echotomographic system (NEMIO SSA-550A, Toshiba Medical System Corporation, Tokyo) with an electrical linear transducer (mid frequency of 7.5 MHz).

The carotid IMT defined by Pignoli et al. was measured as the distance from the leading edge of the first echogenic line to the leading edge of the second echogenic line[9]. Measurement of IMT was conducted by two well-trained physician. IMT in internal carotid artery was evaluated in the subjects through examination of the common carotid artery, carotid sinus, and internal carotid artery by ultrasound, and the sites associated with the greatest IMT observed on the left and right were measured to obtain maximum IMT values for comparison, in accordance with Japanese Guidelines for Ultrasonic Assessment of Carotid Artery Disease by the Japan Academy of Neurosonology and other previous reports[10,11].

### Quality of life scoring

In addition, the quality of life (QOL) of the subjects was evaluated by using a Japanese version of the diabetes treatment satisfaction questionnaire (DTSQ) developed by C. Bradley [12]. This questionnaire is intended to elicit responses to each of the 8 questions listed in terms of 6-graded answers, where the higher the scores for questions 1, 4, 5, 6, 7, and 8, the higher the QOL of the respondents, while, in contrast, the higher the scores for questions 2 and 3, the lower the QOL of the respondents. In this study, using the maximum total score of 36 for questions 1, 4, 5, 6, 7 and 8 combined, the treatment groups were compared for changes in the DTSQ scores from baseline.

### Statistical Analysis

Data were expressed by medians (inter quartile range). Statistical analysis was performed on each of the parameters evaluated for each group by using Wilcoxon signed-rank test to see if there was any significant difference in each parameter between baseline and after treatment. In addition, each of the parameters evaluated was examined for percentage change in the 6-month follow-up and compared between the treatment groups by using Wilcoxon rank-sum test. The computer program used for statistical analysis was SPSS ver.15.

The study protocol for the current study was approved by the Institutional Review Board of the Jikei University School of Medicine, and was conducted with informed consent obtained from all participating subjects (Current Controlled Trials number, NCT00348231).

## Results

**Figure 1:**
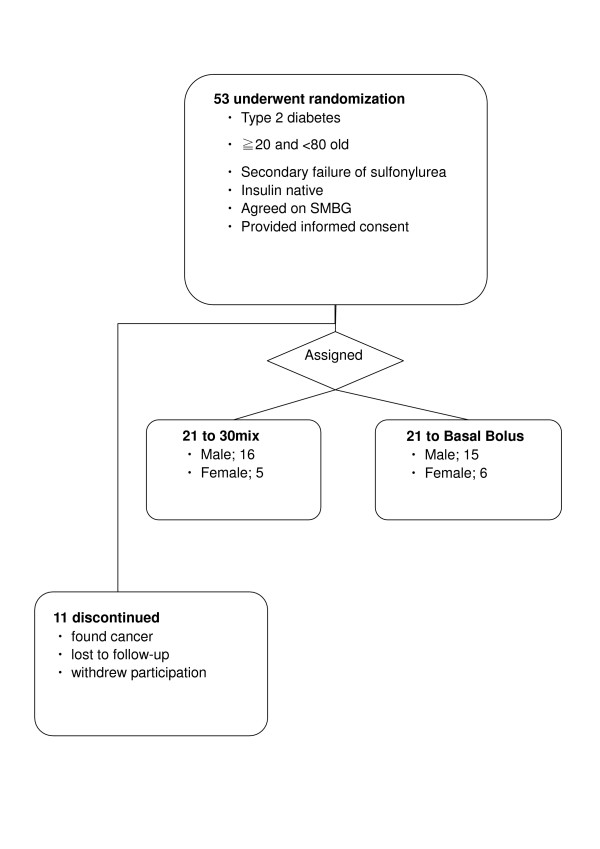
Trial profile.

Of the 53 participants screened, 42 subjects were randomly assigned at the ratio of 1:1 to the 30 Mix group (n = 21) or the BB group (n = 21). Of the 11 subjects who dropped out or were lost to follow-up, 2 subjects had malignancy detected during hospitalization, 5 failed to adhere to the injection instructions, and 4 stopped presenting to the clinic after hospital discharge (Figure 1).

The clinical characteristics of the subjects are given in Table 1. The median (inter quartile range) age of the subjects was 64.5(56.8~71.0) years of age; the ratio of males, 73.8%; the median BMI, 23.7(21.9~26.1) kg/m^2^; the median HbA1c, 9.2(7.9~10.6)%; and the median duration of SU use, 11.0(6.0~15.3) years. There was no significant difference in the distribution of these values between the groups.

**Table 1:** Clinical characteristics of study subjects

	Total	30 mix	Basal Bolus	p
n	42	21	21	
Male(%)	31 (73.8)	16 (76.2)	15 (71.4)	0.73
Age (y)	64.5 (56.8–71.0)	63.0 (55.5–71.0)	65.0 (59.0–70.5)	0.65
BMI(kg/m^2^)	23.7 (21.9–26.1)	23.8 (21.7–26.3)	23.5 (22.4–26.3)	0.79
HbA1c(%)	9.2 (7.9–10.6)	9.3 (8.1–11.3)	8.9 (7.7–10.0)	0.24
Duration of SU drug use (y)	11.0 (6.0–15.3)	11.0 (5.0–16.5)	10.0 (6.5–15.0)	0.76

Data are expressed as frequencies (%), or median (inter quartile range). The sex ratio between the groups were compared using chi-square test. The medians of other data were compared using Wilcoxon rank-sum test. BMI: Body Mass Index. SU: sulfonylurea.

**Figure 2:**
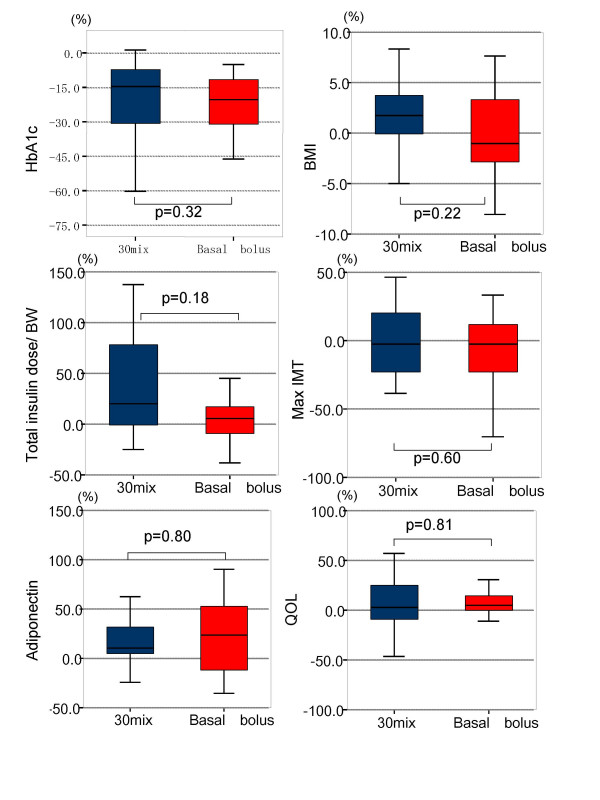
**Median percentage Change from Baseline to 6 months in HbA1c, BMI, Insulin dose/BW, Max IMT, Adiponectin, QOL.** The box-and-whisker plots represented medians and inter quartile ranges and ranges. The medians of each data were compared using Wilcoxon rank-sum test. BMI; body mass index, BW; body weight, Max IMT; maximum intima media thickness, QOL; quality of life.

HbA1c was significantly reduced in both groups after 6 months of insulin therapy compared to baseline (see Additional file 1; 30 Mix;9.3(8.1~11.3) → 7.4(6.9~8.7)%, p < 0.01, vs BB;8.9(7.7~10.0) → 6.9(6.2~7.3)%, p < 0.01), with no significant difference noted in percentage change in HbA1c between the groups (Figure 2; 30 Mix; -14.7(-32.5~-7.5)% vs BB -17.8(-30.1~-11.1)%, p = 0.32).

**Figure 3:**
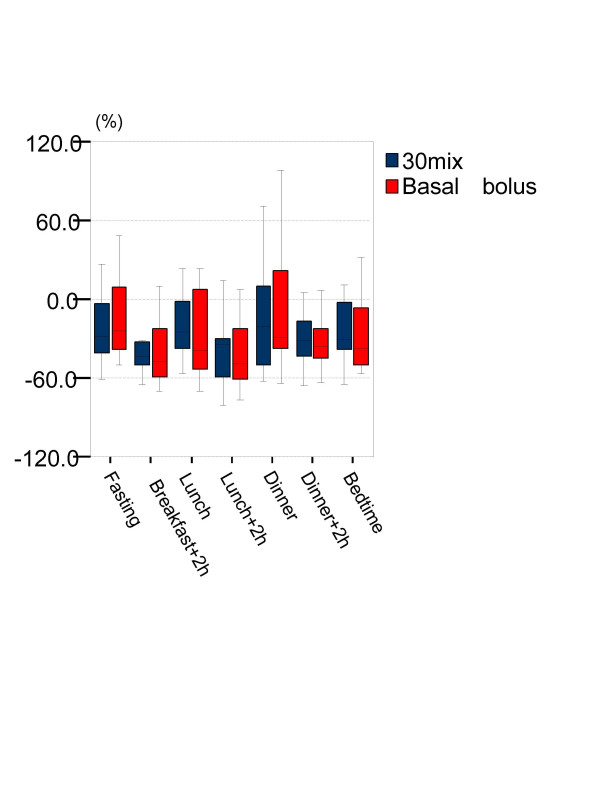
**Median percentage Change from Baseline to 6 months in daily profile of self-measured capillary glucose**. The box-and-whisker plots represented medians and inter quartile ranges and ranges. No significant difference was found between the both group in any time.

There was a significant decrease in daily glycemic profile at all time points except dinner following initiation of insulin therapy in both groups compared to baseline (see Additional file 1), with no significant difference noted in percentage change in glucose levels between the groups (Figure 3).

There was no significant difference seen in percent change in insulin used per kilo of body weight between the groups (Figure 2; 30 Mix; 18.7(-2.4~-58.7)% vs BB; 5.3(-19.0~45.3, p = 0.18). However, the 30 Mix group tended to increase the amount of insulin used comparing with the BB group. While there was a significant increase in the absolute amount of insulin used in the 30 Mix group after 6 months of insulin therapy compared to baseline (see Additional file 1; 30 Mix;0.30(0.17~0.44) → 0.39(0.31~0.42) IU/kg, p = 0.01), there was no significant difference in this parameter in the BB group compared to baseline (see Additional file 1; BB; 0.31(0.24~0.49) → 0.44(0.27~0.53) IU/kg, p = 0.31).

While there was no significant difference in carotid IMT in either group compared to baseline (see Additional file 1; 30 Mix; 1.5(1.2~2.2) → 1.4(1.1~2.1), p = 0.93 vs BB;1.8(1.3~2.5) → 1.8(1.2~2.5)mm, p = 0.50), there tended to be a decrease in percentage change in IMT in the BB group (Figure 2; 30 Mix; -6.7(-25.5~17.3)% vs BB; -4.8(-20.0~11.8)%, p = 0.60).

There was significant difference in adiponectin levels in 30 Mix group between before and after insulin therapy (see Additional file 1; 30 Mix; 7.5(5.3~9.0) → 7.8(6.9~9.5) μg/ml, p = 0.02 vs BB;7.0(5.2~14.5) → 9.0(6.2~14.0) μg/ml, p = 0.16). However, there tended to be an increase in this parameter in both groups, while this was not statistically significant (Figure 2; 30 Mix; 10.3(2.0~37.8)% vs BB; 24.4(-11.6~52.9)%, p = 0.80).

The patient QOL tended to improve after initiation of therapy in both groups, (see Additional file 1; 30 Mix; 26.0(21.5~28.5) → 26.0(21.0~31.5), p = 0.40 vs BB;22.0(18.0~26.5) → 25.0(18.8~29.3), p = 0.06), however no significant difference noted, either, in percentage change in this parameter between the groups (Figure 2; 30 Mix; 3.3(-13.6~26.8)% vs BB; 2.9(-3.8~8.0)%, p = 0.81).

## Discussion

The current study in patients with secondary failure demonstrated that no significant difference was seen in changes in HbA1c or in percentage change in HbA1c after 6 months of therapy between BB and 30 Mix group.

The DCCT [2,3] and the Kumamoto study [4] provided further lines of evidence showing that, in both type 1 and type 2 diabetes, intensive insulin therapy is better able to prevent diabetic complications than conventional insulin therapy, with the added benefit of favorable HbA1c status achieved in patients given intensive insulin therapy.

In contrast to these reports, our study with its focus on HbA1c as the primary indicator of glycemic control showed that comparable results can be obtained both in type 2 diabetic patients given basal-bolus insulin therapy (BB group) and in those given conventional therapy with a biphasic insulin analogue (30 Mix group).

The present study also showed that, while there was no significant difference between two treatment groups in percentage change in HbA1c, however the median HbA1c after 6 months of treatment higher in the 30 Mix group (30 Mix group 7.4(6.9~8.7)% vs BB group 6.9(6.2~7.3)%, p = 0.02). Because HbA1c level of the 30 Mix group tended to be higher than that of the BB group in the study started point (Table 1; 30 mix 9.3 (8.1–11.3)% vs BB 8.9 (7.7–10.0)%, p = 0.24). The mean absolute glucose level after each meal in the 30 Mix group was in excess of 200 mg/dl, suggesting that postprandial glucose levels tended to be higher in the 30 Mix group. Therefore, it is suggested that improving postprandial hyperglycemia is required to achieve greater HbA1c control in the 30 Mix group in our study. In this regard, of note, Monnier et al[13] reported the relative contribution of fasting plasma glucose (FPG) versus postprandial plasma glucose (PPG) to changes in HbA1c as being 30% versus 70% in patients with HbA1c ranging under 7.3%, respectively, while at the same time adding that this ratio comes close to 50% versus 50% in patients with HbA1c ranging between 7.3% and 8.4%. Woerle et al. [14] also showed that glycemic control to FPG 100 mg/dL or lower results in HbA1c 7% or lower being achieved in 64% of patients, while glycemic control to PPG 140 mg/dL or lower results in HbA1c 7% or lower being achieved in 94% of patients.

While there was no significant difference noted between the treatment groups in changes in BMI after initiation of insulin therapy, BMI tended to increase in the 30 Mix group, possibly due to the increase in body weight associated with the amount of insulin use that tended to increase in the 30 Mix group.

In our study, IMT was measured as a surrogate endpoint to examine whether or not insulin therapy might promote atherosclerosis. There was virtually no difference in IMT values from baseline in both the 30 Mix and BB group. However, our study had limitation that the observational period of 6 months might not be long enough to evaluate atherosclerosis.

In this regard, postprandial glucose levels were evaluated as factors linked to IMT. Postprandial hyperglycemia has long been known to be a risk factor for diabetic macrovascular complications [6,7,15-17], and in agreement with this, our study results also demonstrated that daily glycemic profile tended to be higher after lunch in the 30 Mix group after 6 months of insulin therapy than in the BB group (unpublished data), suggesting that more rigorous glycemic control may have contributed to the trend for a decrease in IMT becoming manifest in the 30 Mix group.

Also, adiponectin was evaluated as factors linked to IMT. Adiponectin tended to increase in both groups after initiation of insulin therapy, while there was no significant difference between the two groups. In this regard, IMT and adiponectin are reported to be negatively correlated [18], consistently with the results of the current study.

With regard to the relationship between exogenous insulin and adiponectin *in vivo*, there is one study in pediatric type 1 diabetes reporting an increase in adiponectin 1 month after initiation of insulin therapy [19], while, to date, there is no published study in type 2 diabetes. However, adiponectin secretion is known to be influenced by factors that induce insulin resistance, which include tumor necrosis factor (TNF)-α [20], interleukin (IL)-6 [21], C-reactive protein (CRP) [22], lipid metabolism [23], diet and exercise habits [24,25]. Further research is required to clarify how these insulin resistance-related factors, as they come into play as confounding factors, may behave after initiation of insulin therapy to elucidate the relationship between exogenous insulin and adiponectin.

QOL was shown to improve in both groups after initiation of insulin therapy compared to baseline, with no significant difference shown between the groups. The study subjects appear to have felt not so much stress in starting insulin therapy as satisfaction in being able to achieve better glycemic control, which likely contributed to an increase in their QOL. However, the current study did not include a crossover design, and the subjects stayed on the injection regimen assigned at randomization and never had the opportunity to experience the other injection regimen. Therefore, it was felt that a crossover study might be required to account for differences, if any, in QOL associated with either injection regimen.

After the DCCT and Kumamoto study that demonstrated the efficacy of insulin therapy in type 1 and type 2 diabetes, the advent of biphasic insulin formulations has added to the diversity of insulin regimens available. Thus, there are a number of studies reported that compared insulin therapy with a biphasic insulin analogue and that with an ultra rapid-acting insulin analogue as in the current study. Of these, Joshi et al. [26] reported that HbA1c improved better with twice-daily insulin therapy using a biphasic insulin analogue than with basal-bolus insulin therapy using an ultra rapid-acting insulin analogue, while a recently published report from the 4-T study [27] showed that conventional therapy with a biphasic insulin analogue and basal-bolus therapy with an ultra rapid-acting insulin analogue produce comparable reductions in HbA1c. However, these reports provided very little insight into the impact of insulin therapy on diabetic complications, particularly progression of atherosclerosis. In this regard, ours is the first to demonstrate that there is no significant difference between the insulin regimens compared in their impact on progression of IMT.

In this study, as there was an increase in glucose levels after lunch in some patients given the twice-daily regimen, Further research will be necessary regarding the effectiveness of additional daytime insulin injections or for additional OHA in the individual patient in 30 Mix group.

## Conclusion

This study findings suggest that both basal-bolus therapy with the ultra rapid-acting insulin analogue and conventional therapy with the twice-daily biphasic insulin analogue produce comparable reductions in HbA1c in type 2 diabetic patients with secondary failure, and that given no difference seen between both the regimens in glycemic control, progression of atherosclerosis, and improvement in QOL. The basal-bolus insulin therapy may not be necessarily needed if the type 2 diabetic patients have become secondary failure.

## Authors' contributions

All authors carried out recruitment of patients. YM, NR, NT participated in the design of the study and performed the statistical analysis. All authors read and approved the final manuscript.

## Supplementary Material

Additional file 1 Table2. Outcomes. Data are expressed as median (inter quartile range). The medians of data were compared using Wilcoxon signed-rank test. BG: blood glucose, BMI: body mass index, IMT: intima media thickness, QOL: quality of life. Click here for file
