# Supplementary material for: Prospective randomized study for optimal insulin therapy in type 2 diabetic patients with secondary failure
Source: Cardiovasc Diabetol. 2008 May 29;7:16. doi: 10.1186/1475-2840-7-16 (PMC2442047; doi:10.1186/1475-2840-7-16)
Supplement: Additional file 1 — Table2. Outcomes. Data are expressed as median (inter quartile range). The medians of data were compared using Wilcoxon signed-rank test. BG: blood glucose, BMI: body mass index, IMT: intima media thickness, QOL: quality of life. [file 1475-2840-7-16-S1.doc]

|  | 30mix | | | Basal　Bolus | | |
| --- | --- | --- | --- | --- | --- | --- |
| Month | 0 | 6 | p | 0 | 6 | ｐ |
| HbA1c(%) | 9.3 (8.1-11.3) | 7.4 (6.9-8.7) | <0.01 | 8.9 (7.7-10.0) | 6.9 (6.2-7.3) | <0.01 |
| BG(mg/dl) |  |  |  |  |  |  |
| Fasting | 184 (162-234) | 138 (115-162) | <0.01 | 164 (138-176) | 129 (112-149) | 0.03 |
| Breakfast + 2h | 301 (276-344) | 179 (154-162) | <0.01 | 264 (239-299) | 157 (113-202) | <0.01 |
| Lunch | 252 (228-286) | 149 (92-170) | <0.01 | 244 (196-286) | 126 (98-161) | <0.01 |
| Lunch + 2h | 298 (249-355) | 217 (196-280) | <0.01 | 242 (201-297) | 140 (127-221) | <0.01 |
| Dinner | 197 (172-226) | 137 (114-183) | 0.11 | 181 (125-221) | 134 (119-157) | 0.10 |
| Dinner + 2h | 293 (234-325) | 200 (161-267) | 0.01 | 247 (201-307) | 162 (128-191) | <0.01 |
| Bedtime | 261 (240-304) | 183 (158-243) | <0.01 | 238 (184-292) | 162 (128-191) | <0.01 |
| BMI(kg/m^2^) | 23.8 (21.7-26.3) | 23.8 (22.2-26.2) | 0.20 | 23.5 (22.4-26.3) | 23.5 (22.0-26.7) | 0.96 |
| Daily insulin dose (IU/kg) | 0.30 (0.17-0.44) | 0.39 (0.31-0.42) | 0.01 | 0.31 (0.24-0.49) | 0.44 (0.27-0.53) | 0.31 |
| IMT(mm) | 1.5 (1.2-2.2) | 1.4 (1.1-2.1) | 0.93 | 1.8 (1.3-2.5) | 1.8 (1.2-2.5) | 0.50 |
| Adiponectin(μg/ml) | 7.5 (5.3-9.0) | 7.8 (6.9-9.5) | 0.02 | 7.0 (5.2-14.5) | 9.0 (6.2-14.0) | 0.16 |
| QOL scoring | 26 (22-29) | 26 (21-26) | 0.40 | 22 (18-27) | 25 (19-28) | 0.06 |
